# Supplementary material for: Small molecule modulation of protein corona for deep plasma proteome profiling
Source: Nat Commun. 2024 Nov 7;15:9638. doi: 10.1038/s41467-024-53966-z (PMC11544298; doi:10.1038/s41467-024-53966-z)
Supplement: Supplementary file 1 — Supplementary Information [file 41467_2024_53966_MOESM1_ESM.pdf]

Small molecule modulation of protein corona for deep plasma proteome profiling

Ali Akbar Ashkarran[1#], Hassan Gharibi[2#], Seyed Amirhossein Sadeghi[3], Seyed Majed Modaresi[4], Qianyi Wang[3], Teng-Jui Lin[5], Ghafar Yerima[6], Ali Tamadon[6], Maryam Sayadi[7], Maryam Jafari[8], Zijin Lin[1], Danilo Ritz[9], David Kakhniashvili[10], Avirup Guha[11], Mohammad R.K. Mofrad[6], Liangliang Sun[3], Markita P. Landry[5,12,13], Amir Ata Saei[4,14]* and Morteza Mahmoudi[1]*

[1]Department of Radiology and Precision Health Program, Michigan State University, East Lansing, MI 48824, USA

[2]Division of Chemistry I, Department of Medical Biochemistry and Biophysics, Karolinska Institutet, Stockholm, Sweden

[3]Department of Chemistry, Michigan State University, 578 South Shaw Lane, East Lansing, MI 48824, United States

[4]Biozentrum, University of Basel, 4056 Basel, Switzerland

[5]Department of Chemical and Biomolecular Engineering, University of California, Berkeley, Berkeley, CA 94720, USA

[6]Molecular Cell Biomechanics Laboratory, Departments of Bioengineering and Mechanical Engineering, University of California Berkeley, Berkeley, CA 94720, USA

[7]Department of Biomedical Engineering, Michigan State University, East Lansing, MI 48824, USA

[8]Division of ENT Diseases, Department of Clinical Science, Intervention and Technology, Karolinska Institutet, Stockholm, Sweden

[9]Proteomics Core Facility, Biozentrum, University of Basel, 4056 Basel, Switzerland

[10]Proteomics and Metabolomics Core Facility, University of Tennessee Health Science Center, Memphis, TN, USA

[11] Cardio-Oncology Program, Medical College of Georgia at Augusta University, Augusta, GA 30912, USA

[12]Department of Neuroscience, University of California, Berkeley, Berkeley, CA 94720, USA

[13]Chan Zuckerberg Biohub, San Francisco, CA 94063, USA

[14]Center for Translational Microbiome Research, Department of Microbiology, Tumor and Cell Biology, Karolinska Institutet, Stockholm 17165, Sweden

[#]equal contribution

*Corresponding authors: (A.A.S.) amir.saei@unibas.ch; (M.M.) mahmou22@msu.edu

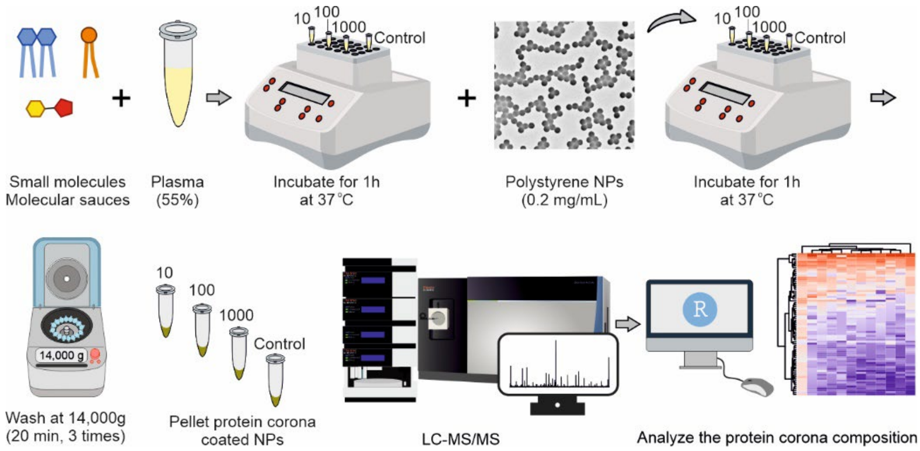

Supplementary Figure 1. The overall workflow of the study. After exposing small molecules to human plasma, NPs were incubated with the treated or untreated plasma, purified, isolated, and used for analysis of the protein corona profile on the surface of the NPs using bottom-up and/or top-down LC-MS/MS.

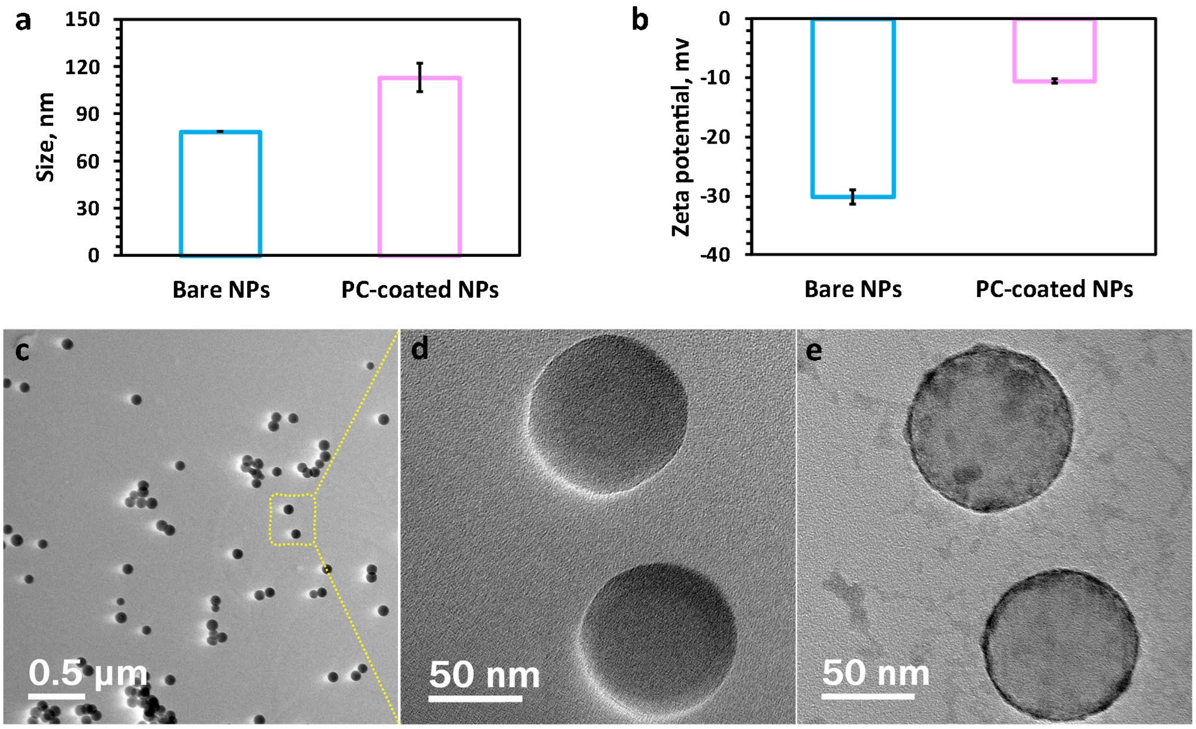

Supplementary Fig. 2. Characterizations of the bare NPs and untreated protein corona coated NPs. (**a**) and (**b**), DLS and zeta potential analysis of bare NPs and untreated protein corona–coated NPs respectively, (**c**), and (**d**) TEM images of bare polystyrene NPs, and (**e**) TEM image of protein corona–coated NPs, as representative. The polydispersity index (PDI) of bare and protein corona-coated NPs were found to be 0.023 and 0.214, respectively.

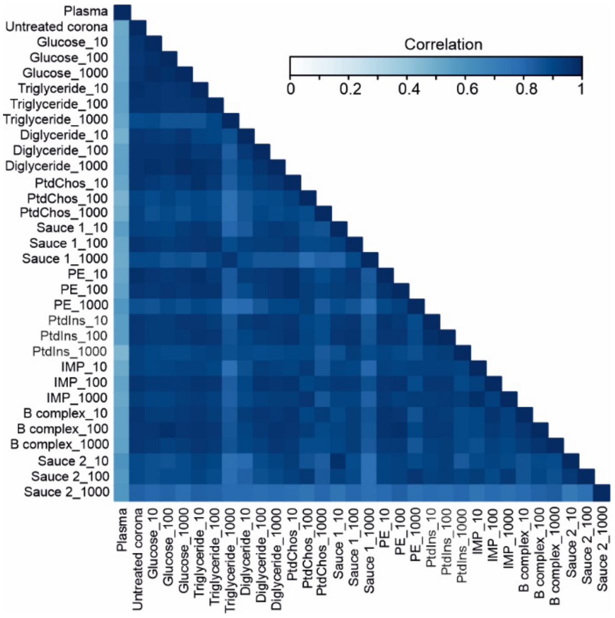

Supplementary Fig. 3. Correlation of plasma proteome profiles. Pearson correlation of the 117 shared proteins across all the samples (10-1000 µg/ml).

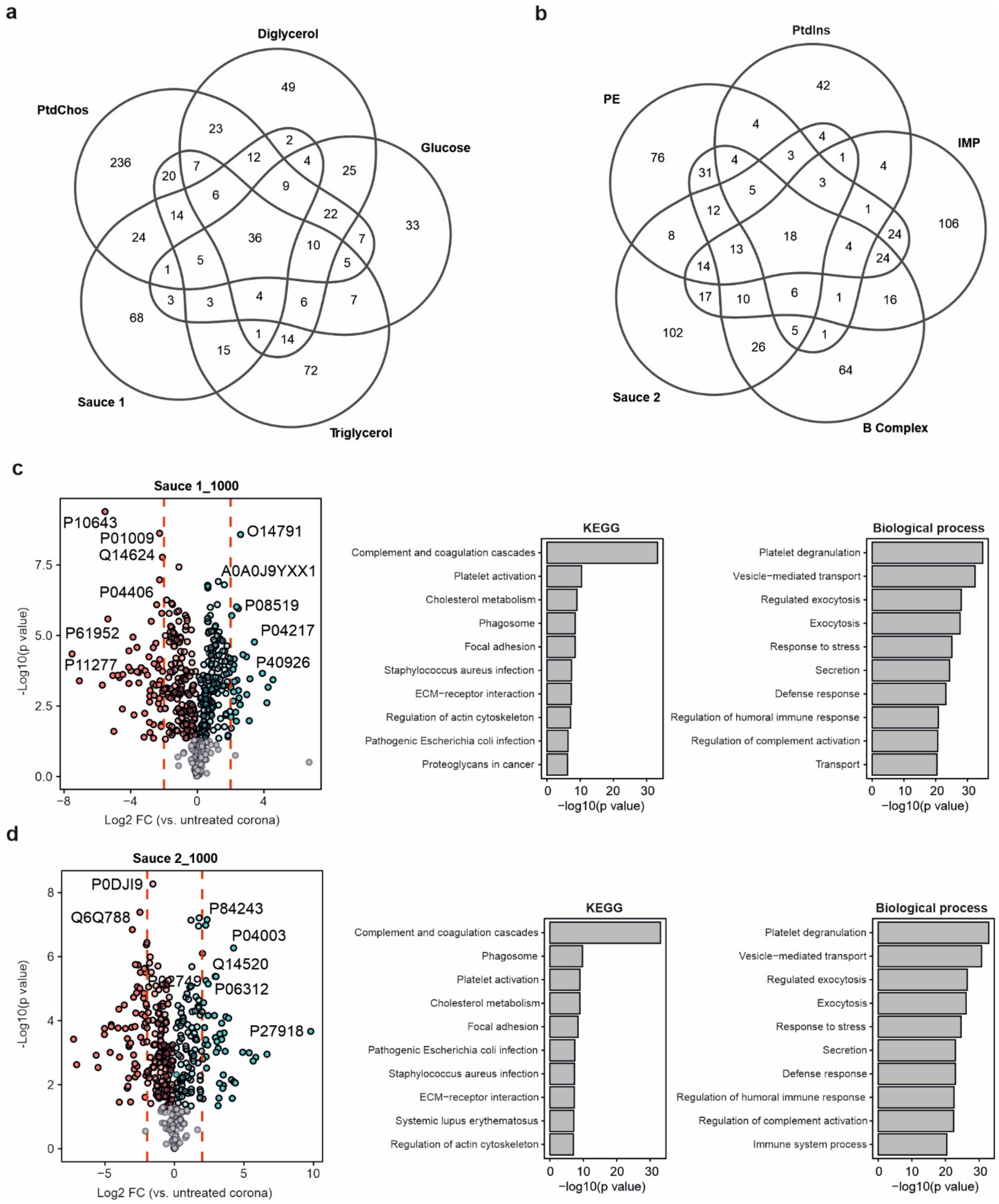

Supplementary Fig. 4. The molecular sauces enrich or deplete specific proteins. **a-b,** The number of unique proteins that were quantified in a given group which were not quantified in the plasma or with bare NPs. **c-d,** The enriched and depleted proteins for molecular sauce 1 and 2 in comparison to the untreated protein corona are shown (left panels). Respective pathway analysis was performed for all the depleted and enriched proteins (right panels). Significance was calculated using Student's t-test. The cyan and red proteins are more and less represented in the sauce samples compared to untreated corona, respectively.

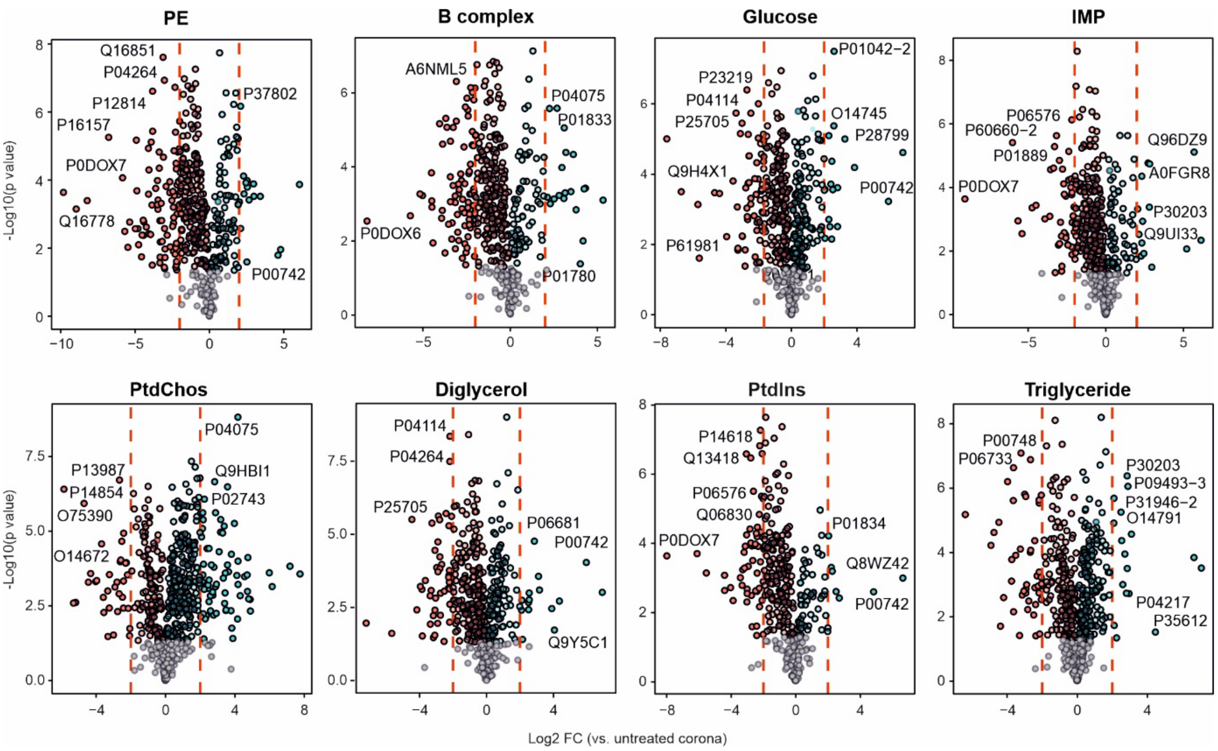

Supplementary Fig. 5. The enrichment and depletion of specific proteins (only those shared) by spiking small molecules in NP protein corona vs. the abundance of proteins in the untreated NP protein corona. Only the results for the highest concentration of each small molecule (1000 µg/ml) are shown.

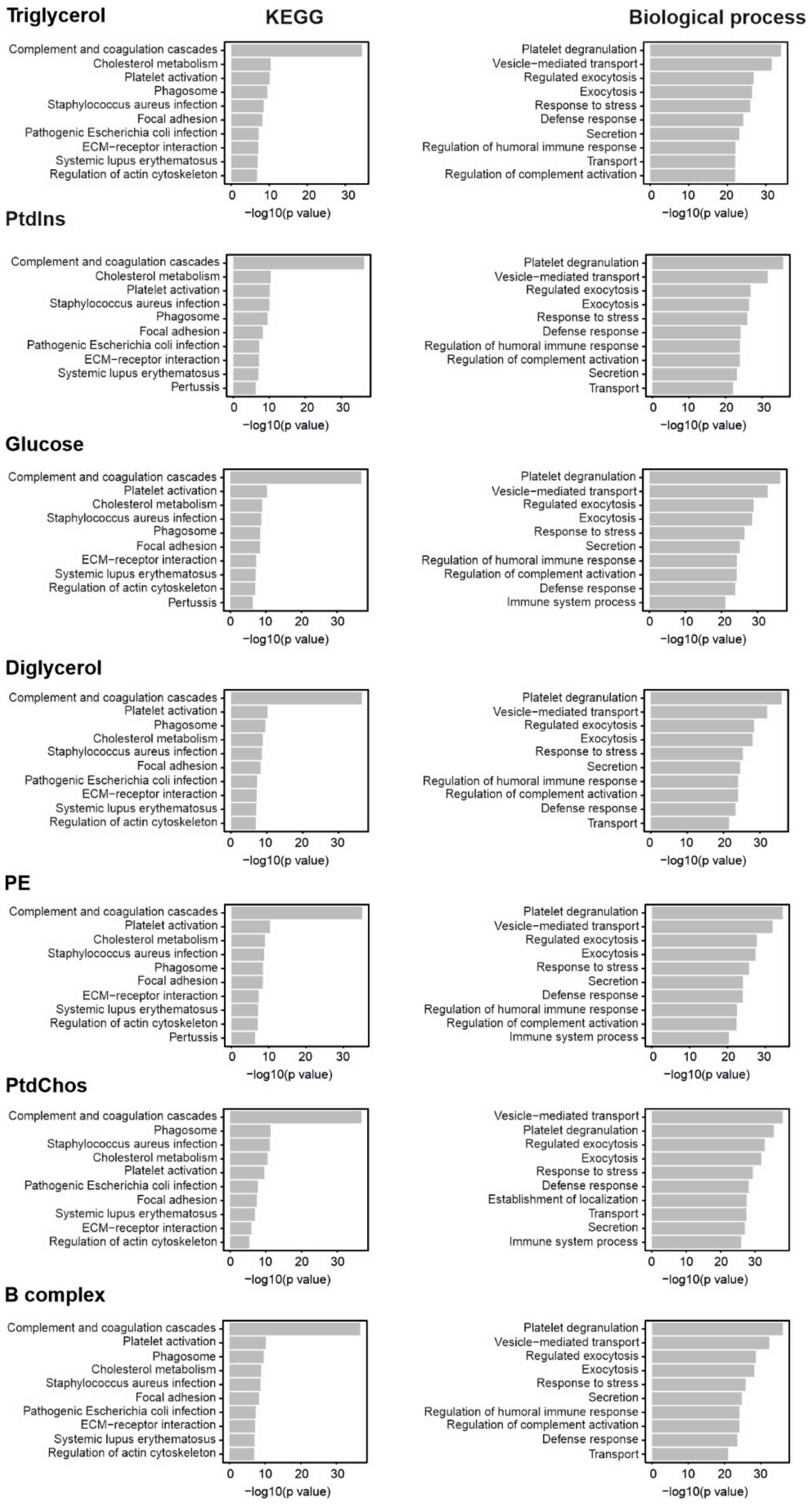

Supplementary Fig. 6. Pathway enrichment for all significantly enriched and depleted proteins for each small molecule cumulatively across all concentrations. KEGG and biological processes are shown.

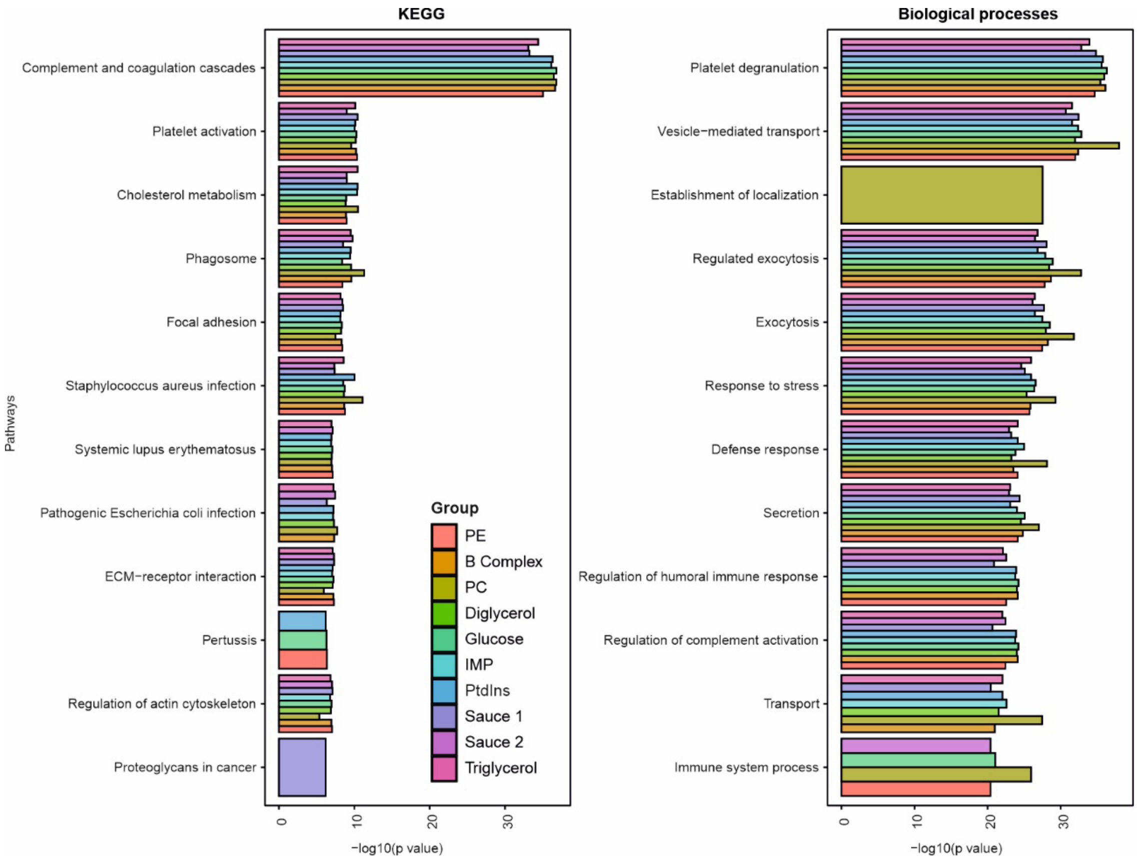

Supplementary Fig. 7. The combined enrichment plot for all small molecules and molecular sauces vs. untreated protein corona, cumulatively across all concentrations.

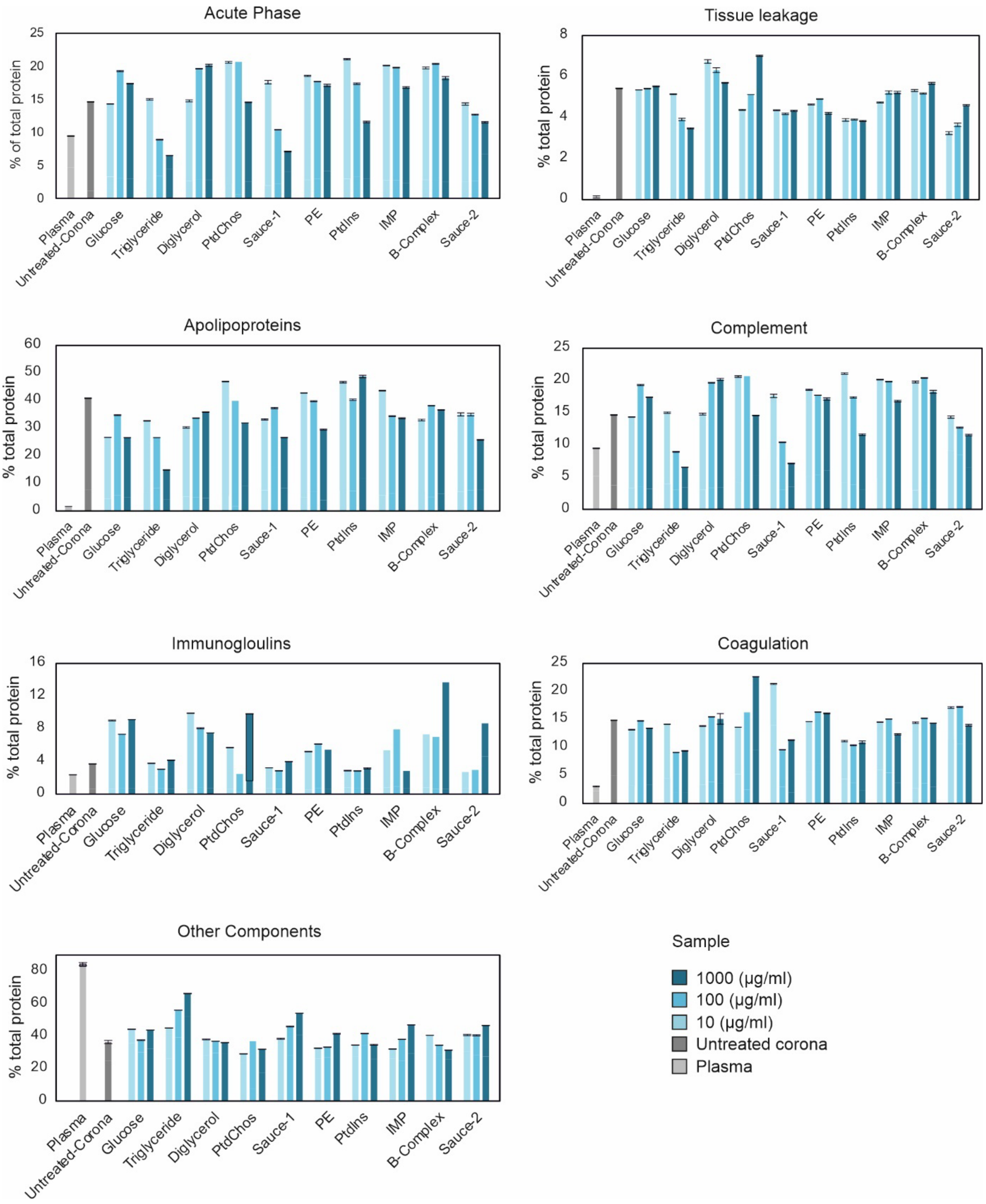

Supplementary Fig. 8. Classification of quantified protein corona of various small molecules according to their physiological functions.

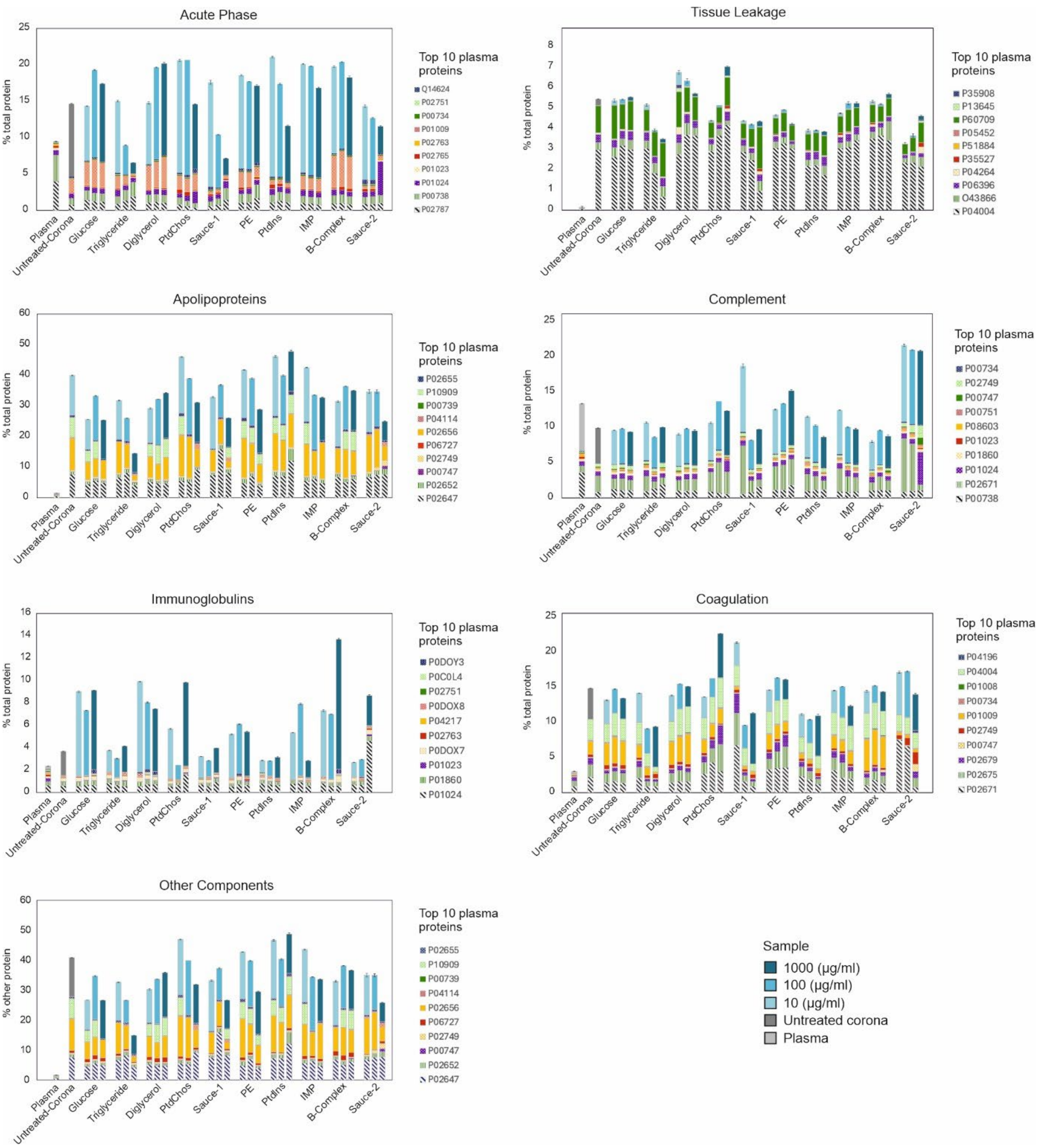

Supplementary Fig. 9. Variations of top 10 plasma proteins across protein corona compositions of various small molecules according to their physiological functions.

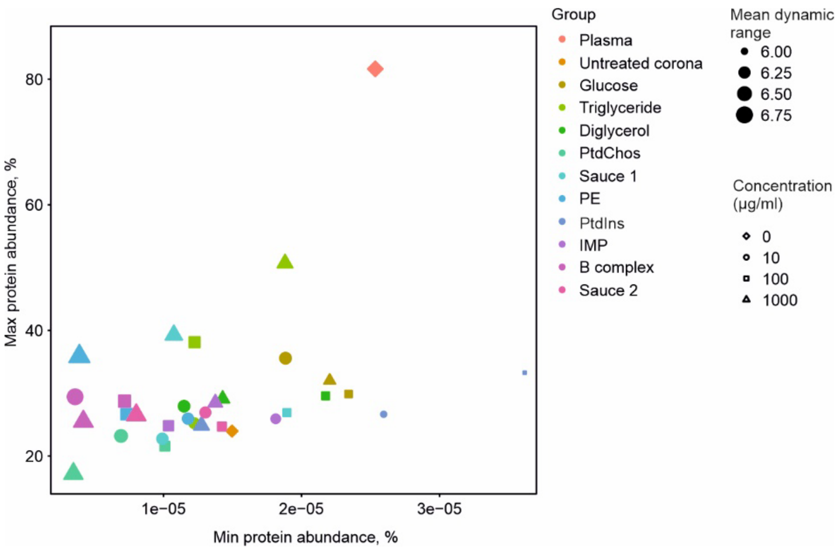

Supplementary Fig. 10. The impact of spiking small molecules on the proteome dynamic range. The dynamic range (order of magnitude) of the proteomics analysis for different samples is shown.

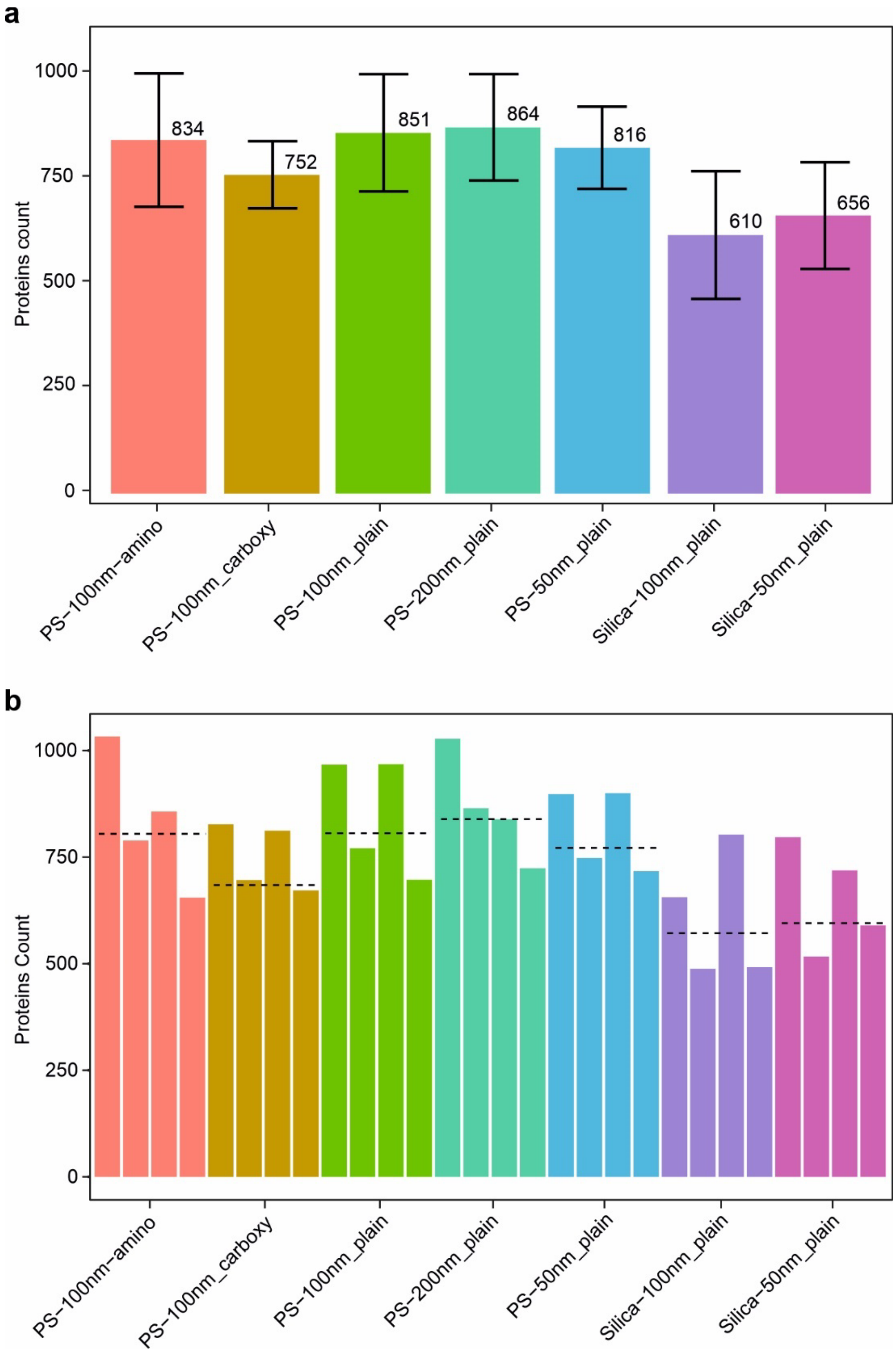

Supplementary Fig. 11. The impact of NPs of different composition, size and charge. **a,** The association of using various NPs with different sizes and charges with average protein count using plasma from 4 individual donors. Error bars represent mean±SD. **b,** same as panel a, but protein count shown for the 4 individual donors separately for each NP type. The line represents the average protein count.

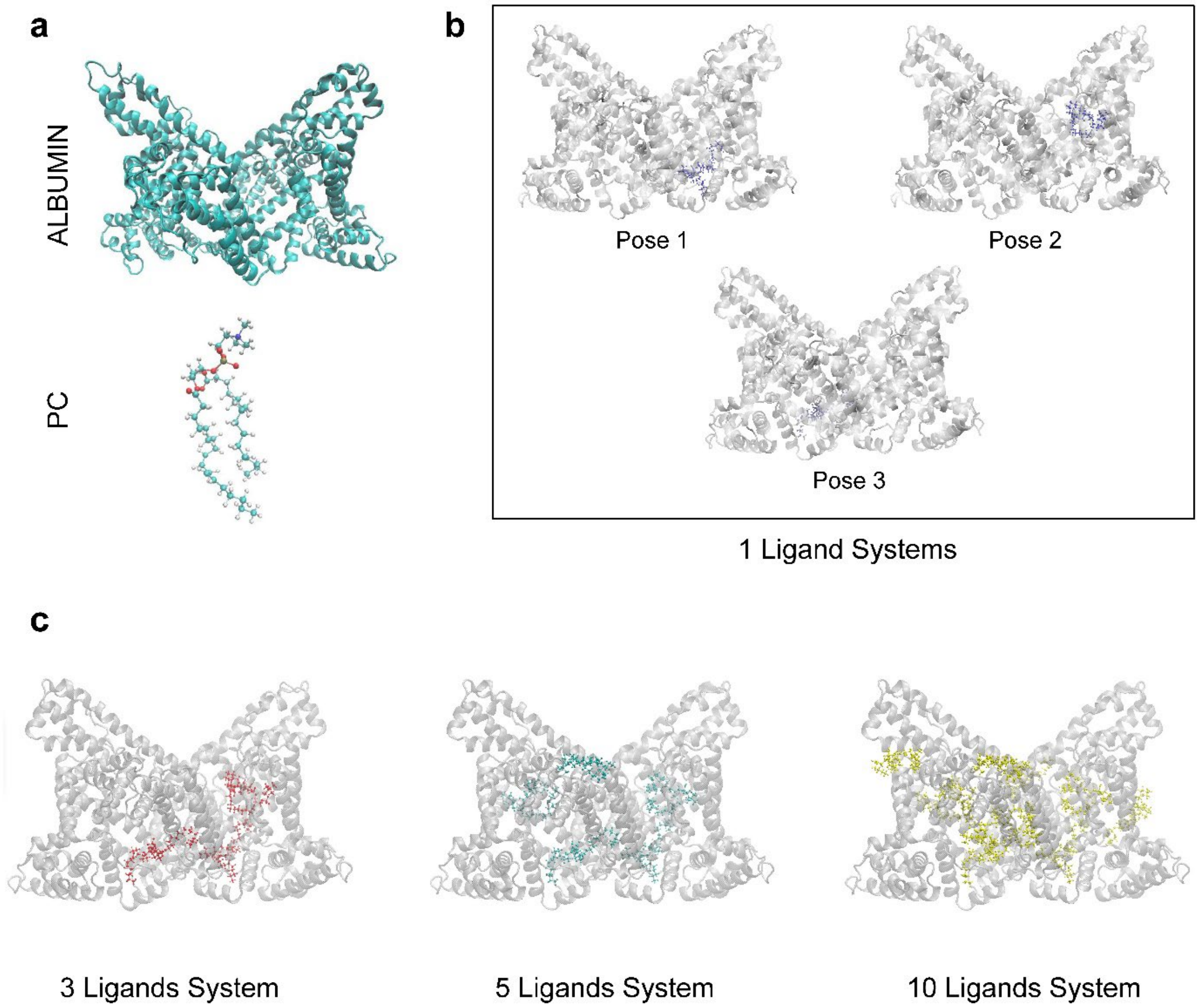

Supplementary Fig. 12. All-atoms molecular dynamics simulations components. a, Crystal structure of albumin and representation of a single PtdChos (PC) molecule. **b,** Crystal structure of albumin showcasing the 1 ligand systems composed of the top 3 favorable binding sites for PtdChos as determined by molecular docking. **c,** Crystal structure of albumin illustrating the 3, 5, and 10 ligands systems which represent the the top 3, 5, and 10 most favorable binding sites for PtdChos respectively.

Supplementary Tables

Supplementary Table 1. The number of quantified proteins as well as the SD and CV of the number of quantified proteins for plasma, untreated protein corona and protein corona treated with various small molecules and molecular sauces (3 technical replicates)

**Group**	**Concentration**	**Protein count**	**SD**	**CV (%)**
Plasma	NA	218	0.58	0.27
Untreated corona	NA	681	2.64	0.39
Glucose	10	615	6.11	0.99
Glucose	100	583	2.52	0.43
Glucose	1000	568	5.69	1.00
Triglyceride	10	682	3.61	0.53
Triglyceride	100	622	3.61	0.58
Triglyceride	1000	632	4.36	0.69
Diglycerol	10	665	2.31	0.35
Diglycerol	100	596	2.31	0.39
Diglycerol	1000	572	4.36	0.76
PtdChos	10	607	5.86	0.97
PtdChos	100	474	NA	NA
PtdChos	1000	897	3	0.33
Sauce 1	10	510	1.53	0.30
Sauce 1	100	473	1.73	0.37
Sauce 1	1000	622	1.53	0.25
PE	10	576	1	0.17
PE	100	603	2.64	0.44
PE	1000	578	1	0.17
PtdIns	10	434	3	0.69
PtdIns	100	397	2.52	0.63
PtdIns	1000	410	5.13	1.25
IMP	10	550	2.08	0.38
IMP	100	717	1.53	0.21
IMP	1000	538	4.93	0.92
B complex	10	613	3.21	0.52
B complex	100	533	5.69	1.07
B complex	1000	552	4	0.72
Sauce 2	10	459	7.02	1.53
Sauce 2	100	567	5.51	0.97
Sauce 2	1000	558	2.65	0.47

Supplementary Table 2. The number of quantified proteins as well as the SD and CV of the number of quantified proteins for plasma, untreated protein corona and protein corona treated with various concentrations of PtdChos (3 technical replicates)

**Group**	**Concentration**	**Average protein count**	**SD**	**CV (%)**
Choline	100	818	1.15	0.14
Choline	500	836	2.52	0.30
Choline	1000	957	3.06	0.32
Choline	10000	856	3.06	0.36
Untreated corona	NA	617	12.12	1.97
Plasma	NA	294	1.73	0.59

Supplementary Table 3. The number of quantified proteins as well as the SD and CV of the number of quantified proteins for protein corona treated with PtdChos and incubated with different NPs (plasma from 4 different donors)

**Sample**	**Protein count**	**Average protein count**	**SD**	**CV (%)**
PS_100nm_Amino_PtdChos_donor1	1033	834	157.26	18.87
PS_100nm_Amino_PtdChos_donor2	789	834	157.26	18.87
PS_100nm_Amino_PtdChos_donor3	857	834	157.26	18.87
PS_100nm_Amino_PtdChos_donor4	655	834	157.26	18.87
PS_100nm_Carboxylated_PtdChos_donor1	827	752	79.08	10.52
PS_100nm_Carboxylated_PtdChos_donor2	696	752	79.08	10.52
PS_100nm_Carboxylated_PtdChos_donor3	812	752	79.08	10.52
PS_100nm_Carboxylated_PtdChos_donor4	672	752	79.08	10.52
PS_100nm_PtdChos_donor1	967	851	138.16	16.24
PS_100nm_PtdChos_donor2	771	851	138.16	16.24
PS_100nm_PtdChos_donor3	968	851	138.16	16.24
PS_100nm_PtdChos_donor4	697	851	138.16	16.24
PS_200nm_PtdChos_donor1	1028	864	125.33	14.51
PS_200nm_PtdChos_donor2	865	864	125.33	14.51
PS_200nm_PtdChos_donor3	839	864	125.33	14.51
PS_200nm_PtdChos_donor4	724	864	125.33	14.51
PS_50nm_PtdChos_donor1	898	816	96.96	11.89
PS_50nm_PtdChos_donor2	748	816	96.96	11.89
PS_50nm_PtdChos_donor3	900	816	96.96	11.89
PS_50nm_PtdChos_donor4	717	816	96.96	11.89
Si_100nm_PtdChos_donor1	656	610	150.75	24.72
Si_100nm_PtdChos_donor2	488	610	150.75	24.72
Si_100nm_PtdChos_donor3	803	610	150.75	24.72
Si_100nm_PtdChos_donor4	492	610	150.75	24.72
Si_50nm_PtdChos_donor1	797	656	125.877	19.19
Si_50nm_PtdChos_donor2	517	656	125.87	19.19
Si_50nm_PtdChos_donor3	719	656	125.87	19.19
Si_50nm_PtdChos_donor4	590	656	125.87	19.19
