# Supplementary material for: Small molecule modulation of protein corona for deep plasma proteome profiling
Source: Nat Commun. 2024 Nov 7;15:9638. doi: 10.1038/s41467-024-53966-z (PMC11544298; doi:10.1038/s41467-024-53966-z)
Supplement: Supplementary file 2 — Description of Additional Supplementary Files [file 41467_2024_53966_MOESM2_ESM.pdf]

Description of Additional Supplementary Files

File Name: Supplementary Data 1
Description: Proteomics data on plasma, untreated protein corona, 8 small molecules and 2 molecular sauces

File Name: Supplementary Data 2
Description: The enriched and depleted proteins for different small molecules and molecular sauces in comparison to the untreated protein corona

File Name: Supplementary Data 3
Description: Proteomics data on the protein coronas on polystyrene NPs when exposed to different concentrations of PtdChos

File Name: Supplementary Data 4
Description: DIA LC-MS data on a single plasma sample along with untreated NP protein corona, and protein corona on NPs when exposed to PtdChos at 1000 µg/µl

File Name: Supplementary Data 5
Description: Proteomics data obtained from different NPs with plasma from 4 donors incubated with PtdChos

File Name: Supplementary Data 6
Description: Top-down proteomics data
